# Up-Cycling of LCD Glass by Additive Manufacturing of Porous Translucent Glass Scaffolds

**DOI:** 10.3390/ma14175083

**Published:** 2021-09-05

**Authors:** Arish Dasan, Paulina Ożóg, Jozef Kraxner, Hamada Elsayed, Elena Colusso, Luca Grigolato, Gianpaolo Savio, Dusan Galusek, Enrico Bernardo

**Affiliations:** 1 Centre for Functional and Surface-Functionalized Glass, Alexander Dubček University of Trenčín, Študentská 2, 911 50 Trenčín, Slovakia; arish.dasan@tnuni.sk (A.D.); paulina.ozog@tnuni.sk (P.O.); jozef.kraxner@tnuni.sk (J.K.); dusan.galusek@tnuni.sk (D.G.); 2 Ceramics Department, National Research Centre, Cairo 12622, Egypt; elsisy_chem@yahoo.com; 3 Department of Industrial Engineering, University of Padova, 35131 Padova, Italy; elena.colusso@unipd.it (E.C.); luca.grigolato@phd.unipd.it (L.G.); 4 Department of Civil, Environmental and Architectural Engineering: Dept. ICEA, University of Padova, 35131 Padova, Italy; gianpaolo.savio@unipd.it; 5 Joint glass centre of the IIC SAS, TnUAD, and FChFT STU, FunGlass, Alexander Dubček University of Trenčín, 911 50 Trenčín, Slovakia

**Keywords:** glass recycling, LCD glass, additive manufacturing, direct ink writing, scaffolds

## Abstract

Additive manufacturing technologies, compared to conventional shaping methods, offer great opportunities in design versatility, for the manufacturing of highly porous ceramic components. However, the application to glass powders, later subjected to viscous flow sintering, involves significant challenges, especially in shape retention and in the achievement of a substantial degree of translucency in the final products. The present paper disclosed the potential of glass recovered from liquid crystal displays (LCD) for the manufacturing of highly porous scaffolds by direct ink writing and masked stereolithography of fine powders mixed with suitable organic additives, and sintered at 950 °C, for 1–1.5 h, in air. The specific glass, featuring a relatively high transition temperature (T_g_~700 °C), allowed for the complete burn-out of organics before viscous flow sintering could take place; in addition, translucency was favored by the successful removal of porosity in the struts and by the resistance of the used glass to crystallization.

## 1. Introduction

A common perception concerning glass is its infinite recyclability [1], supported by its remarkable durability (preventing any degradation over time) and by the characteristic transition to the liquid state (‘glass transition’, T_g_) at moderate temperatures. Such perception, however, is not confirmed by industrial practice. The sustainability of recycling in a strict sense, i.e., reuse of cullet for the manufacturing of the original articles (‘closed-loop recycling’), is controversial, e.g., when considering the relatively scarce saving in ‘embodied energy’ (energy that must be committed to create a mass of usable material), passing from mineral feedstock to recycled material [2].

A fundamental limiting factor in the efficiency of glass recycling is the difficulty in the separation of glass from other materials, such as polymers and metals [3]. Contaminants have a negative impact on the quality of the original glass articles, expressed by defects such as stones and streaks [4] or deviations from the chemical composition. These are absolutely intolerable in specific fields, such as glasses for pharmaceutical containers or optical devices, such as liquid crystal displays (LCD).

The reuse of LCD glass in new products (‘open-loop recycling’) is multiform. Kim et al. extensively studied waste LCD glasses as a raw material for manufacturing of commercial soda-lime silicate [5], glass wool [6] and E-glass [7] products, or as a substitute for feldspar in porcelain sanitary-ware [8] and ceramic tiles [9]. Several patented works [10] concerned the utilization of LCD glass waste in concrete, water treatment, sound-absorbing, and thermal insulating materials, bricks, hygienic pottery, and cement clinker. Yoo and You [11] found that the addition of LCD glass powder as a filler in ultra-high-performance concrete (UHPC) could improve the pull-out resistance of the steel fibers from the concrete. Tsai et al. (2019) [12] developed mesoporous alumino-silicate nanocomposites, from thin-film transistor LCD glass waste after alkali activation and acid leaching, exhibiting an excellent absorption capacity toward metal ions in acidic conditions (pH = 1.5–7.0).

Some investigations were specifically concerned with the viscous flow sintering of LCD glass with limited additives [13,14]. Viscous flow sintering may be considered as a fundamental milestone for sustainable reuse of waste glass, since it is feasible at far lower temperatures than those required for remelting [15,16,17,18]. In fact, glass powders may form a ‘pyroplastic’ mass, when heated above the so-called dilatometric softening point (T_d_), i.e., slightly higher than the glass transition temperature (T_g_) [19]. The dilatometric softening point (corresponding to a viscosity of 10^11.5^ Pa·s [19]) represents a minimum temperature for glass sintering, that could be adopted in hot pressing operations [20]. Pressure-less sintering of glass is generally feasible at temperatures 50–100 °C above T_d_ [21], but still well below ‘Littleton’ softening point, corresponding to gross viscous flow (viscosity of approximately 10^6.6^ Pa·s [19]).

A key for sustainable glass sintering is represented by the definition of products of adequate value to compensate for the expenses associated with the thermal treatment. Glass foams are probably the most established products of this kind. The first procedures for the manufacturing of foams (dating back to nearly one century ago), relying on gas bubbling in molten glass [22], were successfully replaced by glass sintering with concurrent gas evolution from foaming agents. For LCD glasses, the manufacturing of foams (with the support of additives such as MnO_2_ [13] and Na_2_CO_3_ or CaCO_3_ [14]), however, cannot be considered as the only up-cycling option. In fact, foams do not completely valorize some key features, such as the high resistance to devitrification and the remarkable transparency.

**Figure 1:**
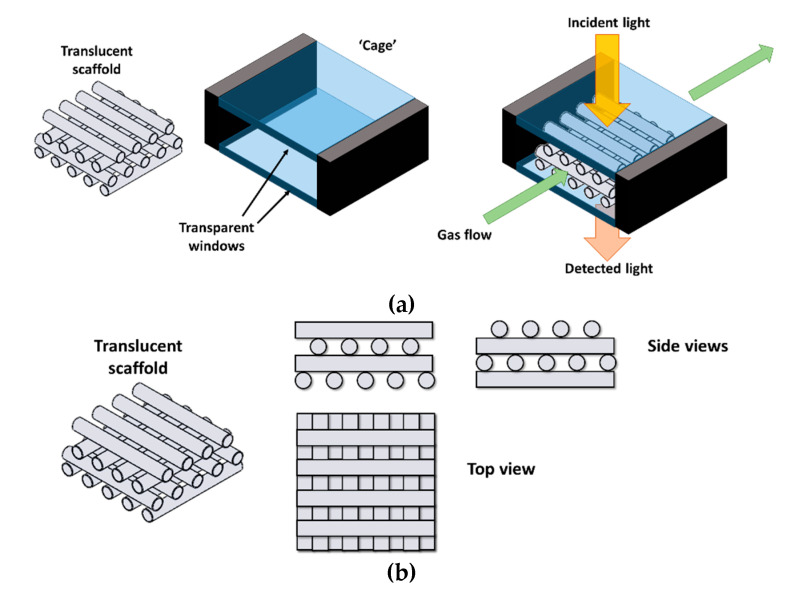
(**a**) Scheme for optical sensing device; (**b**) top and side views of a translucent scaffold.

The present paper aims at disclosing the potential of discarded LCD glass (at least of its cleanest fractions) in the manufacturing of three-dimensional translucent scaffolds, designed as supports for photocatalysts or as a part of optical sensors, based on inorganic coatings that change optical properties (e.g., refractive index) when interacting with noxious gases [23]. As illustrated by Figure 1a, gas could flow through a ‘cage’, consisting of two transparent windows, sandwiching a translucent scaffold: gas may flow through the porosity of the scaffold, while light may travel from the upper window to the lower window only by passing through the struts. If the struts are coated with a sensitive thin layer, the presence of noxious gases in the gas stream would be detected from changes in the light transmission from the upper to the lower window. The struts must be placed in a way that ‘masks’ the lower window from the upper one (see Figure 1b), preventing direct transmission.

The obtainment of highly porous scaffolds with suitable morphology motivated the adoption of an additive manufacturing approach, i.e., direct ink writing (DIW) of pastes of LCD glass and organic binders, known for its simplicity and wide range of material processability [24]. The relatively high characteristic temperatures of the glass and its resistance to crystallization proved to be key features for successful removal of any contamination from the binders and the development of nearly pore-free struts upon firing. These features also constituted a valid starting point for preliminary studies on a more advanced additive manufacturing technology, such as masked stereolithography (MSLA) of glass powders mixed with photocurable binder of glass powders mixed with photocurable binder. Although subject to future refinement, the obtained results are remarkable for their simplicity, since they involve the sintering of relatively coarse-grained commercial glass, instead of silica glass nano-particles [25], complex precursors (Si−, P− and B-alkoxides) [26], or even glass melts [27], used in pioneering studies on transparent 3D-printed glass components. 

## 2. Materials and Methods

### 2.1. Preliminary Characterizations

Alkali-free glass (SiO_2_: ≥55 wt%, Al_2_O_3_: 15%, B_2_O_3_: 10%, CaO: 10%, SrO: 5%; BaO: 1%) used for liquid crystal and OLED displays was provided in the form of large sheets by NEG (Nippon Electric Glass Co., Ltd., Ōtsu, Japan). The glass was reduced into fine powders, with a size below 38 μm, by manual crushing of sheets into coarse fragments, dry ball milling, and sieving. 

A dilatometric analysis (402E Netzsch Gerätebau GmbH, Selb, Germany) was performed on a glass fragment, while differential thermal analysis (DTA/TGA, Netzsch Gerätebau GmbH, Selb, Germany) was performed on fine powders. A heating rate of 10 °C/min was adopted for both tests. The viscous flow sintering of cylindrical pellets of fine glass powders (pressed at 40 MPa) was monitored by hot stage microscopy (HSM) apparatus (Leitz, Wetzlar, Germany), operating with a heating rate of 5 °C/min, up to 1100 °C.

### 2.2. Scaffolds from DIW

LCD glass powders (total solid load of 75 wt%) were mixed with an organic binder solution based on isopropyl alcohol (2-propanol, Sigma-Aldrich, Germany). The solution was prepared by dissolving 2 g of polyvinyl butyral (Butvar B-98, Sigma-Aldrich, Germany) and 1 g of polyethylene glycol (PEG, Mn 950-1.050, Sigma-Aldrich, Germany) in 20 mL of alcohol under magnetic stirring, at 300 rpm. The mixture was stirred for 1 h, to ensure complete dissolution of components and prepare a clear liquid. 

The glass suspension in alcoholic solution (the ‘ink’) was first mixed manually and then placed in a planetary mixer (Thinky Are-250, Intertronics, Kidlington, UK), operating at 1200 rpm, for 3 min. Subsequently, the ink was placed into a syringe and centrifuged at 2000 rpm, for 3 min, for degassing. The syringe containing the ink was positioned in a printer (Delta Wasp 20 × 40 Turbo 2, Wasp S.r.l., Massa Lombarda, Italy) and connected to compressed air through a pressure regulating valve. The ink was extruded through a nozzle with a diameter of 400 µm or 800 µm (Nordson EFD, Westlake, OH, USA). The printing process was carried out in air (i.e., without any support from a liquid bath). 

Porous lattice structures, with a simple grid, were printed layer-by-layer (for a total of 4–8 layers) by direct extrusion of the developed ink (direct ink writing, DIW); the spacing between parallel extruded filaments and the layer thickness were set as equal to the nozzle size (in agreement with the scheme in Figure 1b). The green printed structures were strong enough to be manually removed from the printing platform, after drying in air at room temperature. Some printing experiments were dedicated to the preparation of samples for bending strength determinations, consisting of bars (after firing) with a cross-section of about 4.6 mm × 2.1 mm.

### 2.3. Scaffolds from MSLA 

Some experiments involved a second suspension of LCD glass powders in organic medium, aimed at the printing of scaffolds by masked stereolithography instead of DIW. Fine glass powders were suspended in a commercially available photocurable acrylic polymer (FunToDo Standard Blend, Lumi Industries S.r.l., Montebelluna, Italy), already comprising a suitable photo-initiator and photo-absorber, at a solid loading of 55 wt%. The mixture was first homogenized at 2000 rpm for 10 min, using the planetary mixer, and then printed using an MSLA machine (Original Prusa SL-1, Prusa Research a.s., Prague, Czech Republic) operating in the visible light range (λ = 405 nm). The layer thickness was set to 50 µm, combined with an exposure time of 15 s/layer. 

After cleaning in an ultrasonic bath with isopropanol for 3 min, the samples were subjected to a secondary curing step in a UV curing chamber (operating wavelength λ = 365 nm, Robot Factory S.R.L., Mirano, Italy) for 15 min. MSLA enabled the printing of lattices and gyroids, in the form of cubic blocks with dimensions of about 10 mm × 10 mm × 10 mm. The adopted geometrical models (STL, Standard Triangulation Language) were derived from a preliminary computational study by the Rhinoceros 6 program package (Robert McNeel & Associates, Seattle, WA, USA), according to the approach described by Savio et al. [28].

### 2.4. Firing and Final Characterizations

Due to the different nature of organic additives, DIW-printed and MSLA-printed samples were subjected to specific debinding programs, based on previous experiments [29]. While DIW samples were treated at 650 °C for 3 h, at a heating rate of 1 °C/min, MSLA samples were treated at 550 °C for 3 h, at a heating rate of 0.2 °C/min. All samples were then sintered at 950 °C for 90 min, using a heating rate of 5 °C/min.

The bulk density was computed from the weight-to-volume ratios of the regular printed blocks after careful determinations of weights and dimensions of the samples by analytical balance (precision of 0.0001 g) and a digital caliper, respectively. The apparent and true densities of various samples were measured by He gas pycnometry (Micromeritics AccuPyc 1330, Norcross, GA, USA), applied on samples both in bulk and powder forms. Morphological and microstructural characterizations were performed by optical stereomicroscopy (AxioCam ERc 5s Microscope Camera, Carl Zeiss Microscopy, Thornwood, New York, NY, USA) and scanning electron microscopy (FEI Quanta 200 ESEM, Eindhoven, The Netherlands). The mineralogical analysis of LCD glass before and after sintering was conducted by X-ray diffraction (XRD; Bruker AXS D8 Advance, Bruker, Germany).

Bars from DIW were subjected to 4-point bending tests (24 mm lower span, 8 mm upper span), at room temperature, employing a Galdabini Quasar 25 UTM material testing machine (Galdabini S.p.a., Cardano al Campo, Italy) operating at a cross-head speed of 1 mm/min. Each data point represents the average value of at least 8 individual tests. The compressive strength of MSLA scaffolds was measured employing the mechanical testing machine specified above, operating at a cross-head speed of 0.5 mm/min. Each data point represents the average value of at least 5 individual tests.

The total transmittance spectrum of DIW-processed sample (4 layers) was collected using a UV-VIS-NIR spectrophotometer (Jasko V570) equipped with an integrating sphere (ISN-470). Measurements were conducted in the 300–800 nm range, by using a data pitch of 1 nm and a spectral bandwidth of 10 nm to collect a significant area of the three-dimensional structure.

## 3. Results and Discussion

### 3.1. Preliminary Sintering Studies

**Figure 2:**
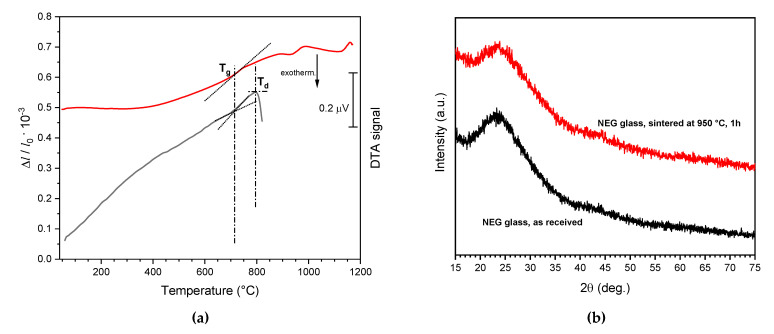
(**a**) DTA and dilatometry plots of the studied LCD glass; (**b**) X-ray diffraction analysis of LCD glass in the as-received state and after sintering at 950 °C (5 °C/min heating rate, 1 h holding time).

Figure 2a provides fundamental information concerning the sintering of the adopted LCD glass. Firstly, the relatively high transition temperature (T_g_~700 °C), poorly visible from the DTA plot (from fine powders) but clearly shown by the dilatometry plot (from a glass fragment), favored the sintering of translucent components. The high T_g_ prevented the risk of sintering glass particles before the completion of the burn-out of organic binders. Furthermore, no crystallization exothermic effect could be detected in the typical temperature range for pressure-less viscous flow sintering (as mentioned above, corresponding to T = T_d_ + (50–100) °C = ~850–900 °C).

No crystallization effect could actually be noted even at higher temperatures (e.g., T = T_d_ + 150–950 °C). In other words, viscous flow sintering of LCD glass could take place without the formation of crystal inclusions, which could increase the viscosity and prevent full densification and lead to opaque products after cooling. The remarkable resistance to crystallization of LCD glass is further testified by the results of X-ray powder diffraction analysis shown in Figure 2b, revealing no obvious difference between the original LCD glass fragments and the pellets of pressed powders sintered at 950 °C, for 1 h (5 °C/min heating rate).

**Figure 3:**
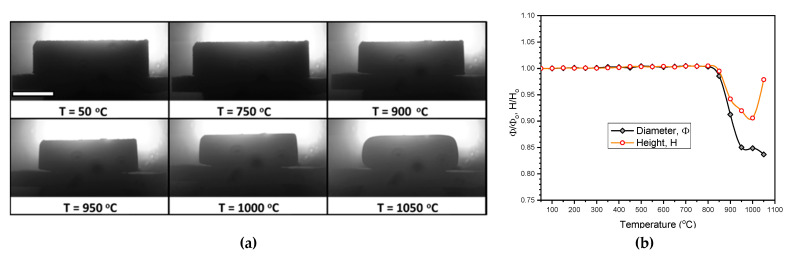
Heating microscopy study of a cylindrical pellet of pressed LCD glass powder: (**a**) evolution of morphology (scale bar = 5 mm); (**b**) changes in relative diameter and relative height.

Furthermore, 950 °C could actually be interpreted as the maximum sintering temperature for LCD glass. As shown by the heating microscopy images (Figure 3a), the edges of a cylindrical pellet of pressed glass powder became more rounded above 950 °C. The shrinkage in both height and diameter was quite uniform in the temperature interval 850–950 °C (Figure 3b), whereas at 1050 °C the shape was completely altered (Figure 3a, bottom right).

### 3.2. DIW Experiments

**Figure 4:**
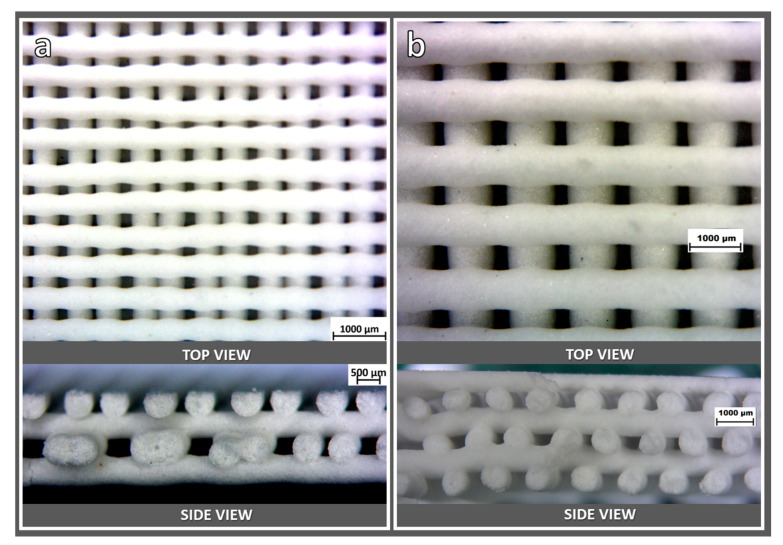
Optical stereomicroscopy top and side views of ‘green’ scaffolds from LCD glass, obtained by extrusion through 400 (**a**) and 800 (**b**) µm nozzles.

The printing accuracy was conditioned by the choice of nozzle size and spacing between filaments. As shown in Figure 4a, printing with the 400 µm diameter nozzle resulted in scaffolds with non-uniform spacing already in the green state, possibly due to vibrations of the printing head. On the contrary, the nozzle with the diameter of 800 µm allowed for a more accurate control of the stacking of filaments, as demonstrated in Figure 4b. In particular, Figure 4b (bottom section) illustrates the fulfillment of the stacking conditions defined in Figure 1b.

**Figure 5:**
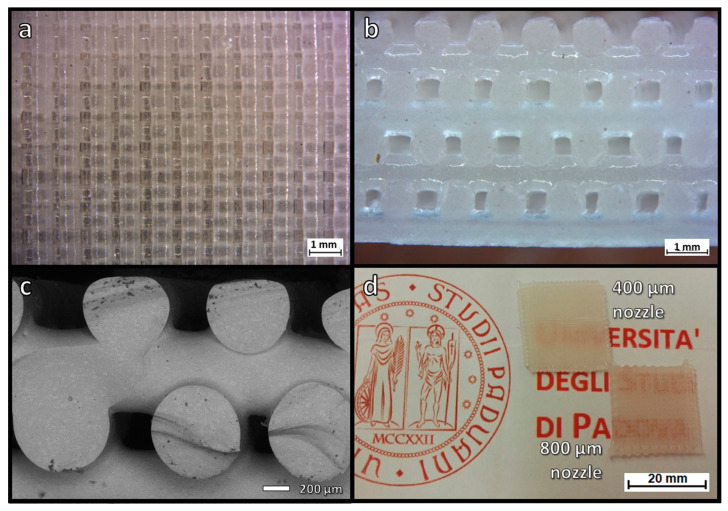
Microstructural details of translucent scaffolds from LCD glass: (**a**,**b**) optical stereomicroscopy top and views of a scaffold from 800 µm nozzle (8 layers); (**c**) scanning electron micrograph of the fracture surface of a scaffold from 800 µm nozzle (4 layers); (**d**) visual comparison of ‘tiles’ from the overlapping of 4 layers.

The uniformity of scaffolds printed with the 800 µm nozzle was also confirmed after firing at 950 °C, as shown by the optical images in Figure 5a,b. As demonstrated by the side view (Figure 5b), the filaments had an excellent interpenetration. The residual porosity was also limited, even for thick samples (from the overlapping of up to 8 layers, Figure 5b). This was further confirmed by scanning electron microscopy of fracture surfaces (Figure 5c). The higher uniformity of scaffolds from wider nozzles, compared to scaffolds from the 400 µm nozzle is finally illustrated by Figure 5d, showing two ‘tiles’ resulting from the stacking of only 4 layers. The stochastic defectivity of the scaffold with finer filaments enhanced the scattering of light. On the contrary, the scaffold printed with the 800 µm nozzle was nearly transparent, despite much higher overall thickness (~2.5 mm against ~1.3 mm).

The physical and mechanical properties of the scaffold printed with the 800 µm nozzle (4 layers) are summarized in Table 1. The final porosity was not particularly abundant (P~35 vol%) but almost completely open. Contrary to the geometrical density, the apparent density determined by He pycnometry of scaffolds was very close to the true density (density of solid LCD glass), measured by He pycnometry of LCD glass powders. The closed porosity did not exceed 0.6 vol%. The porosity and the consequent stress concentration did not cause any substantial degradation of mechanical properties, especially considering the following ‘materials selection index’:

I = σ_f_^2/3^/ρ (1)

where σ_f_ is the failure stress (bending strength, also known as ‘modulus of rupture’) and ρ is the geometrical density. Bars designed to resist a specific applied bending moment may be lighter as the index I increases [30]. It could be noted that the effect of stress concentration was limited, since the tested bars had filaments oriented longitudinally on the tensile side, but the I value of DIW-processed scaffolds (~9 MPa^2/3^·cm^3^/g) was undoubtedly excellent. The achieved index, in fact, approaches that of dense glasses and glass-ceramics (ρ = 2.5–3 g/cm^3^), having a bending strength in the order of 80–100 MPa [31].

**Figure 6:**
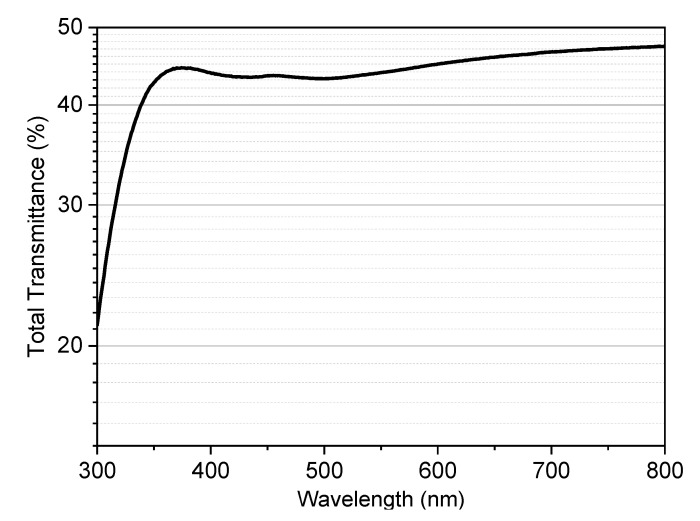
Total transmittance spectrum of a ‘tile’ from the overlapping of 4 layers (800 µm).

The high translucency of the printed scaffolds (4 layers) was further verified by a simple optical test (Figure 6). The total transmittance of a ‘tile’ (bottom right corner of Figure 5d) exceeded 40% in the whole visible range. Such a level is reputed adequate in the perspective of depositing optically active coatings, as mentioned previously [23].

### 3.3. MSLA Experiments

The translucent glass scaffolds produced by DIW fulfilled the design conditions defined in Figure 1. However, the excellent coupling of nearly full strut densification and maintenance of the shape imparted by printing, after firing at 950 °C, motivated further experiments with MSLA. 

Compared to DIW, MSLA generally allows for the definition of very complex shapes, but the adopted starting materials and equipment had to be verified, especially in terms of maintenance of geometries after printing and viscous flow sintering. Some coarsening could be due to light scattering and to poor packing of powders immersed in photocurable liquid.

**Figure 7:**
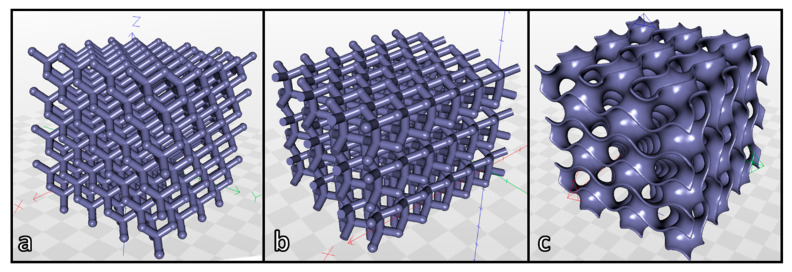
Reference three-dimensional models adopted for MSLA scaffolds: (**a**) diamond symmetry; (**b**) wurtzite symmetry; (**c**) gyroid.

Figure 7 displays the three models adopted for MSLA experiments, all designed for cubic samples having a nominal porosity of 85 vol%, and with similar pore count per side (10 mm long). The different shapes and the (quite high) nominal porosity were selected to highlight possible ‘topological’ contributions to coarsening and tune the strength-to-density ratio. 

The lattices with diamond (Figure 7a) and wurtzite (Figure 7b) geometry share the same tetrahedral coordination of nodal points, but feature a different ‘conformation’. In diamond lattices (resembling the crystal structure of diamond and many semiconductors) the struts (i.e., beams connecting nodal points) are in ‘staggered conformation’, making the structure equivalent along four cubic diagonal directions (cubic symmetry). In wurtzite lattices (resembling the crystal structure of rare hexagonal diamond and of a second series of semiconductors) some struts are placed along an axis of hexagonal symmetry, some others in ‘eclipsed conformation’ [32,33]. Such conformation differences have an impact on printing, with printed layers all intersecting the struts in diamond lattices, or orthogonal to the axis of hexagonal symmetry (vertical axis in Figure 7b) in wurtzite lattices. Gyroids (Figure 7c) rely on a very different concept of porous solid, with ‘channels’ defined by curved membranes [34].

**Figure 8:**
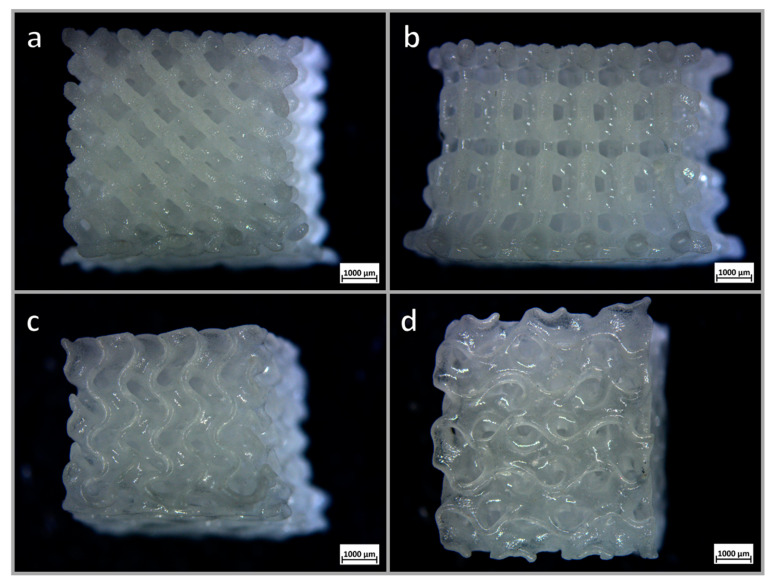
Optical stereomicrographs of MSLA scaffolds after firing at 950 °C: (**a**) diamond cell scaffold; (**b**) wurtzite cell scaffold; (**c**,**d**) gyroid (side and bottom views).

As expected and shown by the side views in Figure 8a–c, the final scaffolds were less porous than the models, but some topological effects could be detected. The diamond geometry (Figure 8a) led to a lower coarsening, with the final porosity approaching 70 vol%. Wurtzite and gyroid models (Figure 8b–d) led to much denser structures. Wurtzite, in particular, exhibited a quite significant distortion due to viscous collapse of the bottom layers (see lower part of Figure 8b). Denser structures exhibited higher compressive strength, up to more than 8 MPa, as reported by Table 1, but in terms of strength-density correlation the lightest samples (diamond cell design) were far more interesting.

For highly porous open-celled structures (such as lattices), the compressive strength, σ_c_, typically follows the well-known Gibson–Ashby (GA) model [35]:

σ_c_ ≈ σ_bend_·0.2·(ρ_rel_)^3/2^ (2)

where ρ_rel_ represents the relative density (ρ_rel_ = 1 − P, where P is the total porosity), whereas σ_bend_ is the bending strength of the solid phase. Reversing the equation and introducing the experimental data of diamond cell samples allowed for an interesting estimation. A compressive strength of 3.7 MPa, with a porosity of 67 vol% (relative density = 0.33), is consistent with a solid phase having a bending strength of about 100 MPa, in excellent agreement with the analysis of strength data of less porous, DIW-processed scaffolds.

**Figure 9:**
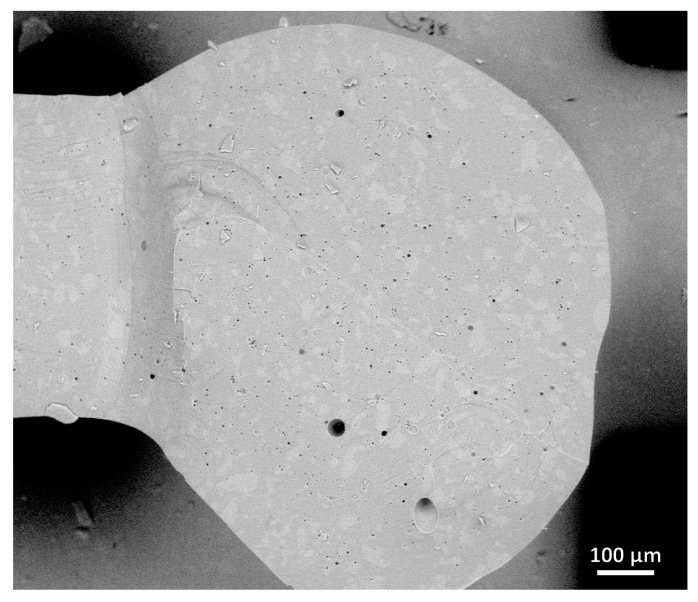
Scanning electron micrograph (backscattered electron image) of fractured wurtzite scaffold.

The densification of struts in MSLA scaffolds was not complete, as shown by the electron microscope image in Figure 9 (note the black dots corresponding to small pores, not observed in Figure 5c). The scattering of light, caused by residual porosity and much higher overall thickness, was more intense than in DIW-processed scaffolds shown in Figure 5d. Figure 9 actually highlights some additional contribution to scattering due from structural inhomogeneity: different tones (also visible in Figure 5c) are attributed to slight variations in the different chemical compositions from the starting glass (backscattered electron emission is susceptible to the atomic weight of the elements). Anyway, the translucency documented in Figure 8d is considered sufficient for the exploitation of scaffolds as supports of photo-catalysts operating in the visible range, e.g., N-doped TiO_2_ [36].

The improvement of the transparency of scaffolds from MSLA will constitute the basis of future work, also considering sintering in vacuum. For the sake of simplicity and sustainability, however, firing in the air would be preferable, especially to control the strut size: scaffolds with thinner struts may be obtained by considering reference models with much more substantial porosity. A ‘calibration’ of the porosity of fired products, on the porosity of as-printed scaffolds (i.e., resin-glass composites), will be needed, with issues represented by adjusting multiple concurrent variables (e.g., solid content, glass granulometry, and printing conditions such as solid content and exposition time, etc.) not yet explored. 

Future optimization studies will undoubtedly involve MSLA, but not exclusively; in fact, in DIW-processing, besides in the formulation of inks, multiple adjustments may arise from modification in the choice of nozzles [37] or slicing sequence [38], as well as in the same concept of machine (classic direct-drive, Cartesian-type printer, instead of the adopted delta-type printer) [39]. Additional efforts are envisaged in the development of analytical models to predict the structural mechanical behavior of printed parts, with fundamental attention to changes in printing patterns, as already done for polymers and polymer-based composites [40,41,42,43].

Although preliminary, the obtained results clearly disclose the potential of additive manufacturing and simple viscous flow sintering for the reuse of clear glasses. Future efforts will also be dedicated to the extension of the approach to other difficult-to-recycle, clear, and crystallization-resistant glasses, such as glasses used for pharmaceutical vials.

## 4. Conclusions

Additive manufacturing technologies, according to the findings of the present investigation, may be seen as novel tools for the valorization of waste glass. In particular, LCD glass is interesting for its relatively high processing temperatures (enabling the complete burn-out of organic binders during firing) and for its resistance to crystallization. Preparation of highly translucent scaffolds, suitable for the manufacturing of innovative gas-sensing devices, is feasible by direct ink writing. Masked stereolithography was confirmed for its potential in the manufacturing of components with more complex shapes, but the inherent coarsening of struts, passing from the geometrical models to the fired products, constitutes a fundamental issue. All LCD glass-based printed structures, although subject to future refinement, already appear adequate for the fabrication of supports for photo-catalysts.

## Figures and Tables

**Table 1:** Physical and mechanical properties of glass scaffolds printed according to different technologies and designs.

Property.	DIW Scaffold (4 Layers)	MSLA Scaffolds		
		Diamond Design	Wurtzite Design	Gyroid Design
Geometrical density, ρ (g/cm^3^)	1.64 ± 0.17	0.80 ± 0.05	0.98 ± 0.02	1.09 ± 0.08
Apparent density (g/cm^3^)	2.49 ± 0.05	2.45 ± 0.05	2.44 ± 0.05	2.42 ± 0.05
True density (g/cm^3^)	2.51 ± 0.05			
Porosity (vol%)	34.7	67.2	59.9	55.2
Bending strength, σ_f_ (MPa)	40.6 ± 14.7	-	-	-
Compressive strength, σ_c_ (MPa)	-	3.7 ± 1.0	6.1 ± 0.5	8.4 ± 1.1

## Data Availability

The data presented in this study are available on request from the corresponding author. The data are not publicly available due to privacy restrictions.
